# Morphotype-specific effector functions of *Cryptococcus neoformans PUM1*

**DOI:** 10.1038/srep23638

**Published:** 2016-03-24

**Authors:** Jan Naseer Kaur, John C. Panepinto

**Affiliations:** 1 Department of Microbiology and Immunology, Witebsky Center for Microbial Pathogenesis and Immunology, Jacobs School of Medicine and Biomedical Sciences, University at Buffalo, the State University of New York., USA

## Abstract

The basidiomycete fungal pathogen *Cryptococcus neoformans* requires the PUF protein, Pum1, for hyphal morphogenesis during sexual development. In this study we found that Pum1 was auto-repressive under growth as yeast, but that auto-repression was relieved during filamentous growth through utilization of an alternative transcription start site driven by the master filamentation regulator Znf2. In addition, Pum1 was required to stabilize the *ZNF2* mRNA through an indirect mechanism suggesting that Znf2 and Pum1 each positively regulate the expression of the other to achieve the filamentous morphotype required for sexual development in *Cryptococcus.*

The PUF (Pumilio and FBF) family of RNA binding proteins regulates cell division, differentiation and development throughout the eukaryotic domain. PUF proteins play a key role in regulating various aspects of embryonic development and gametogenesis in *Drosophila* and *Caenorhabditis elegans*^1^. Via a translation control mechanism, Pumilio also modulates neuron morphogenesis and functions in higher eukaryotes including *Drosophila*, humans and rats^1^,^2^,^3^,^4^. Leaf polarity in *Arabidopsis*, sexual development in the malarial parasite *Plasmodium falciparum,* and organelle biogenesis in *Saccharomyces cerevisiae* constitute some of the other diverse functions performed by PUF proteins^1^,^5^,^6^. Analogous to the known roles of PUF proteins implicated in development of diverse organisms, Pum1 in *C. neoformans* is required for hyphae formation and extension during sexual development after mate recognition and cell fusion^7^.

*C. neoformans* Pum1 is orthologous to both Puf3p of *S. cerevisiae* and Pumilio of *Drosophila melanogaster* with 34% and 46% amino acid sequence identity, respectively. Puf3p binds nearly exclusively to mRNAs that encode mitochondrial proteins in *S. cerevisiae* via a consensus motif of UGUA(CUA)AUA which was most frequently located in the 3′ UTR, and rarely found in the predicted 5′ UTR sequences^8^. A previous study in our lab has shown that Puf4 of *C. neoformans* identifies and binds to its consensus element present in the 5′ UTR of its target *HXL1* transcript^9^. Despite the conservation of Puf3/Pumilio/Pum1 protein and consensus binding element across species, whole genome analyses have revealed a loss of conservation in target mRNAs containing the consensus element^10^. Enrichment of mRNAs encoding mitochondrial proteins was lost with fermentative capacity outside of the *Saccharomycotina*^10^.

The basidiomycete fungal pathogen, *C. neoformans* is unicellular. It has been proposed that most infections by *C. neoformans* occur via the inhalation of spores that, due to their small size, are able to effectively lodge in the alveoli of the lungs^11^. One of the main modes of spore formation is sexual development. This heterothallic fungus has two opposite mating types: *MAT**a*** and *MATα. C. neoformans* can undergo bisexual as well as unisexual reproduction. Same-sex mating is mostly observed in cells of the α mating type in serotype D and is a rare phenomenon for the more virulent serotype A^12^. The opposite sex mating occurs when haploid *MAT**a*** and *MATα* yeast cells undergo cell-cell fusion resulting in hyphae formation^12^. Following this critical morphological switch, karyogamy and meiosis ensue ultimately resulting in basidiospore production. This differentiation process is governed by several transcription factors including the homeodomain proteins Sxi1α and Sxi2**a,** and the zinc finger transcription factor Znf2^7^,^12^. Recently, a genetic screen identified *PUM1* as a post-transcriptional regulator of development in *C. neoformans*^7^.

In this study we demonstrate that Pum1 is auto-regulatory, controlling its own morphotype expression in addition to the downstream process of sexual development. The RNA binding activity of Pum1 was essential for its regulatory function and deletion of the Pum1 consensus binding element in the 5′ UTR of *PUM1* primes cells for mating, suggesting that *PUM1* exerts a repressive role during vegetative growth. The absence of Pum1 severely attenuated the induction of the master filamentation regulator, *ZNF2,* in a synthetic *P*_*GAL7*_*ZNF2* overexpression strain through an indirect mechanism affecting mRNA stability. During hyphal extension, Znf2 drove *PUM1* expression from an alternative transcription start site downstream of the Pum1-binding element, relieving Pum1 auto-repression. These data support a model in which Pum1 is repressive for filamentation during vegetative growth through auto-regulation, and is supportive of filamentation in part through indirect stabilization of *ZNF2,* which in turn drives *PUM1* expression during sexual development.

## Results and Discussion

### Pum1 is auto-regulatory in *C. neoformans*

**Figure 1:**
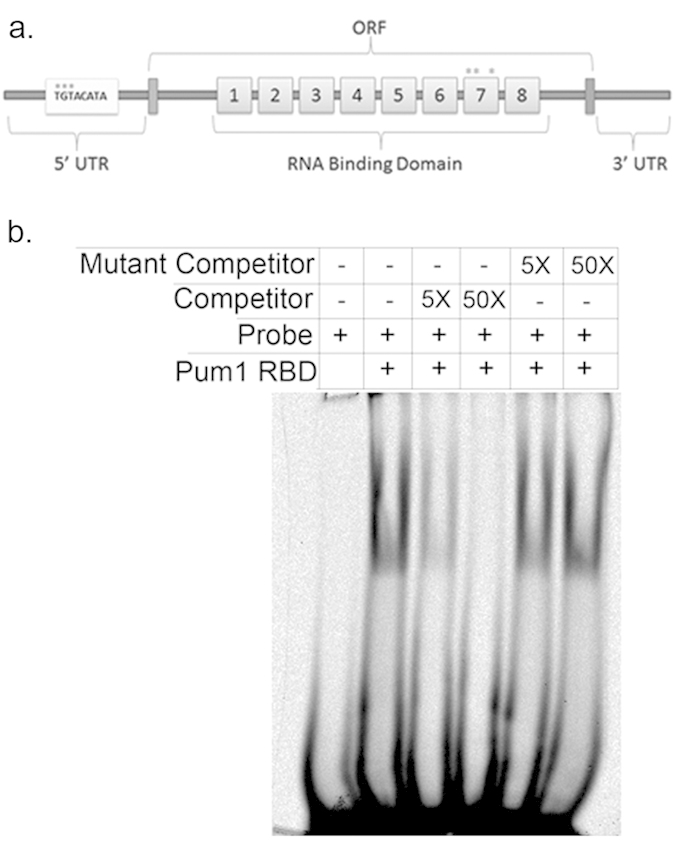
Pum1 binds to its consensus sequence in the 5′ UTR of the *PUM1* mRNA. (**a**) Schematic representation of *PUM1* ORF along with its 5′ and 3′ UTRs. (**b**) Native electrophoretic mobility shift assay demonstrates recombinant Pum1 RNA binding domain binds to the 5′ UTR *PUM1* oligonucleotide. Increasing concentrations of an unlabeled 5′ UTR *PUM1* oligonucleotide competitor was also added to the reaction mixtures to confirm competition. Unlabeled 5′ UTR *PUM1* oligonucleotide with a mutated PUF competitor element was also used in a competition assay to confirm the specificity of the interaction.

Pum1 is required for sexual development in *C. neoformans* after mate recognition and cell fusion, at the point of post-fusion hyphal extension^7^. Very little Pum1 protein is produced in yeast cells, suggesting that there is a morphotype-dependent regulation of Pum1 expression and translation. To begin investigating the mechanism of this morphotype-dependent regulation, we sequenced the ends of the *PUM1* mRNA using rapid amplification of cDNA ends (RACE). Analysis of the sequence obtained from the 5′ and 3′ RACE products amplified from the *PUM1* cDNA revealed the presence of a putative Puf3 consensus binding element (P3E), UGUACAUA, in the 5′ UTR (Fig. 1a).

To determine the ability of the pumilio domain of Pum1 to bind to the P3E in its own 5′ UTR we performed native RNA Electrophoretic Mobility Shift Assay (EMSA). Incubation of recombinant Pum1 RNA binding domain (RBD) protein with the Infrared labeled RNA probe of the *PUM1* 5′ UTR containing the P3E resulted in a relative retardation of oligonucleotide migration compared to the free probe, suggesting binding. Addition of excess unlabeled *PUM1* 5′ UTR oligonucleotide competed for binding. However, when an excess of unlabeled probe in which the invariant UGU was mutated to AAA was added, the shift persisted, suggesting that the binding obtained is specific for the consensus motif (Fig. 1b). This suggests that in *C. neoformans,* Pum1 may regulate the fate of its own message via the presence of a rare 5′ UTR P3E.

### Mutation of the 5′ UTR consensus binding element primes cells for filamentation

**Figure 2:**
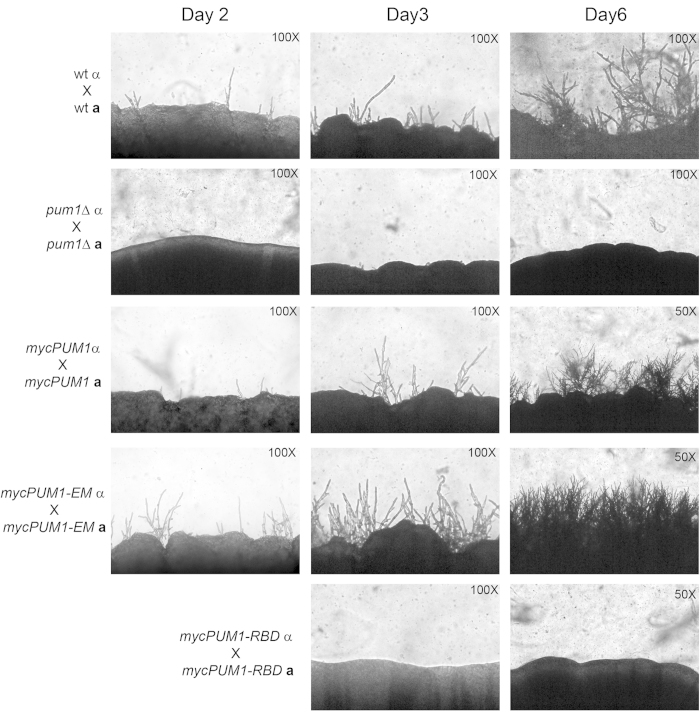
Pum1-P3E interaction is required for both auto-repression and support of filamentation. Equal number of α and **a** mating partners of wild type, *pum1*Δ*, mycPUM1, mycPUM1-EM and mycPUM1-RBD* were cocultured and spotted on to the V8 mating media and incubated in the dark at room temperature. Photographs were taken at the indicated times.

To investigate the biological consequences of Pum1 binding to its own mRNA, we mutated the UGU of UGUACAUA to AAA in the 5′ UTR of a *PUM1-myc* expression construct by site directed mutagenesis. The wild type and mutant constructs were used to complement the *pum1*Δ mutant. Clones with equivalent copy number of the wild type 5′ UTR (*mycPUM1*) or the element mutant 5′ UTR (*mycPUM1-EM*) in both the *MAT**a*** and *MATα* mating types were chosen for further analysis. Crosses of opposite mating-type strains were incubated under mating conditions and monitored for the appearance of filaments by light microscopy. Crosses of the wild type parental strains were included as a control. As it has been suggested previously, *pum1*Δ bilateral cross was defective for filamentation (Fig. 2). We observed that the mating spots of the *mycPUM1-EM* strains were more filamentous compared to those of either the *mycPUM1* complement or the parental cross (Fig. 2). This suggests that binding of Pum1 to its own mRNA is repressive, and that abrogating this interaction primes cells for mating and filamentation.

From the phenotypic consequence of mutating the P3E, we predicted that Pum1 exerts post-transcriptional regulation on its own mRNA. We first set out to verify that the RNA binding activity of Pum1 was directly involved in its role in filamentation. We again transformed the *pum1*Δ mutant with a *mycPUM1* expression construct. In this case, we mutated three residues known to be required for RNA binding in the seventh pumilio repeat of Pum1, S351, N352 and E355, to alanine using site directed mutagenesis, generating both *MAT**a*** and *MATα mycPUM1-RBD* strains. We observed bilateral crosses of the RBD mutants for filamentation, including the wild type parent, *pum1*Δ and the *mycPUM1* complement as controls. Mating assays revealed that the RBD mutants are defective for filamentation compared to the wild type (Fig. 2). These results indicate that the RNA binding activity of Pum1 is essential to its function in promoting post-fusion hyphal extension.

### Mutation of the 5′ UTR Pum1/Puf3 consensus binding element de-represses Pum1/Puf3 translational repression under vegetative growth conditions

**Figure 3:**
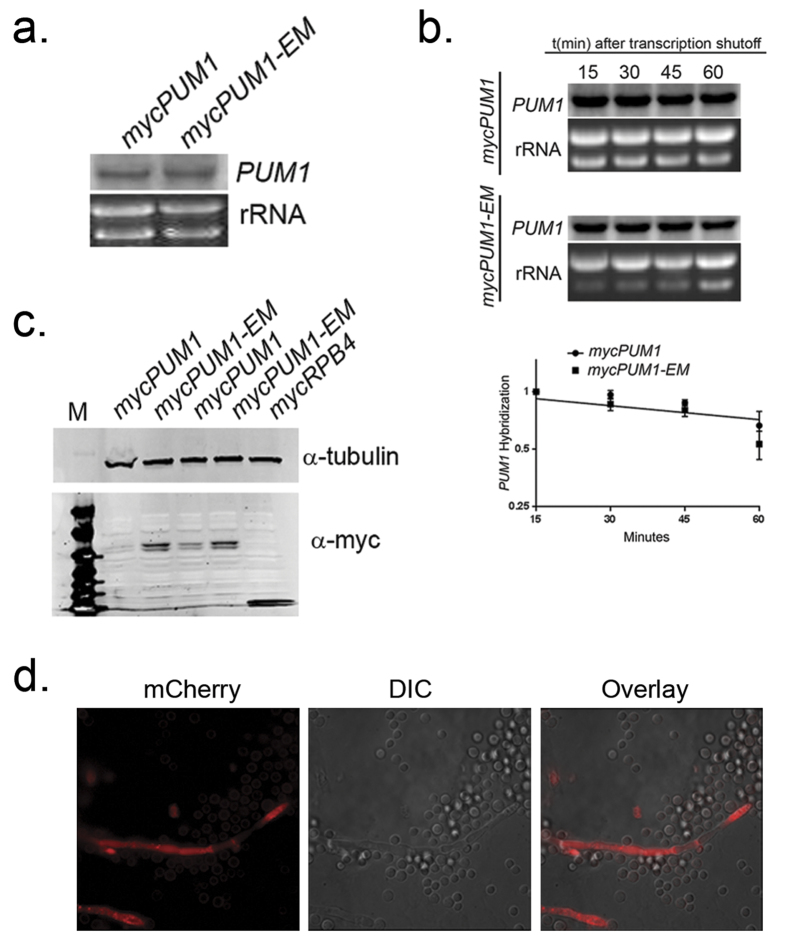
Pum1 post-transcriptionally regulates its own mRNA in a morphotype specific manner. (**a**) Northern analysis of *PUM1* in the *mycPUM1* and *mycPUM1-EM* strains grown to mid-log phase in YPD. (**b**) Mid-log-phase cultures of *mycPUM1* and *mycPUM1-EM* were treated with the transcriptional inhibitor 1,10-phenanthroline and allowed to grow at 30 °C. Aliquots of cells were harvested at 15 min intervals for RNA extraction and subjected to Northern blotting for the detection of *PUM1* transcript. Log 2 plot of relative expression of *PUM1* transcript normalized to the rRNA was used to calculate the mRNA half-life. The graph shown is the representative of three biological replicates. (**c**) Western analysis for mycPum1 in the *mycPUM1* and *mycPUM1-EM* strains grown to mid-log phase in YPD. The membrane was probed with primary mouse anti-myc antibody followed by secondary IR680-conjugated Donkey anti-mouse antibody. (**d**) Fluorescence microscopy of mCh-Pum1 from bilateral mating of mCherry-tagged Pum1 complement strains.

PUF proteins can control the fate of their target mRNAs by regulating mRNA localization, translation and decay^6^. To determine the mechanism by which Pum1 regulates its own mRNA, we compared the abundance and stability of the *PUM1* mRNA and the level of Pum1 protein between the *mycPUM1* and *mycPUM1-EM* strains. Northern blot analysis revealed that the mRNA levels of *PUM1* were not different between the *mycPUM1* and *mycPUM1-EM* strains (Fig. 3a). Unsurprisingly, comparison of *PUM1* mRNA stability between the *mycPUM1* and *mycPUM1-EM* strains revealed no difference between the strains (Fig. 3b). Together this data suggested that the effect of Pum1 on its own transcript was not on the mRNA itself, but rather in a downstream process. To determine if there was a difference at the level of Pum1 protein, we utilized western blot to compare the abundance of Pum1 protein between the *mycPUM1* and *mycPUM1-EM* strains. As demonstrated in Fig. 3c, an increase in Pum1 protein was detected in *mycPUM1-EM* strains compared to the *mycPUM1* strains. A *mycRPB4* strain is included as a positive control. This suggests that during vegetative growth, Pum1 exerts a repressive effect on the translation of its own mRNA through interaction with a P3E in the 5′ UTR. However, when the P3E is mutated, it loses its ability to bind and thus translation repression is relieved.

### De-repression of Pum1/Puf3 is not sufficient to induce filamentation in the absence of mating cues

It was previously reported that Pum1 protein is detected only in filaments^7^. To verify this observation, we complemented our *pum1*Δ mutant with a construct that expresses an mCherry-tagged Pum1 under the control of the *PUM1* promoter in both mating types. Fluorescence microscopy of scrapings from bilateral crosses of the mCherry-tagged Pum1 strains revealed that mCherry fluorescence is only localized to the areas of hyphal growth or septation (Fig. 3d) with surrounding yeast cells exhibiting no observable mCherry fluorescence. The correlation of Pum1 protein expression with filamentation led us to determine if de-repression of Pum1 protein production in yeast cells of the *mycPUM1-EM* strain is sufficient to induce hyphae formation. We spotted *MATα mycPUM1-EM* alone as well as co-cultured it with it’s a mating partner. Despite the de-repression of Pum1 translation that occurs with mutation of the P3E, hyphae formation was only observed in the presence of a mating partner (Supplementary Fig. S1). The phenotype of spots of the α *mycPUM1-EM* alone were afilamentous, suggesting that de-repression of Pum1 is not sufficient to induce filamentation in the absence of opposite mating partner.

### Pum1 indirectly regulates the master filamentation regulator, *ZNF2*, at the level of mRNA stability

**Figure 4:**
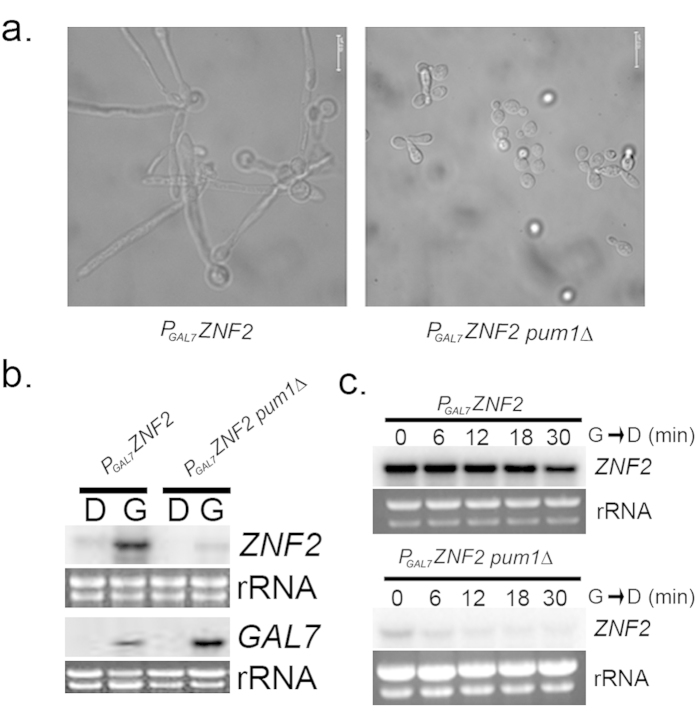
Pum1 modulates the transcript abundance and stability of *ZNF2*. (**a**) Photomicrographs (DIC) of the P_GAL7_*ZNF2* and P_GAL7_*ZNF2 pum1*∆ strains induced for 3 hours. (**b**) Northern analysis of either *ZNF2* or *GAL7* expression in P_GAL7_*ZNF2 and* P_GAL7_*ZNF2 pum1*∆ strains grown to mid-log phase in YPD or induced (YP-GAL) for 3 hours. (**c**) Northern analysis of *ZNF2* stability in the P_GAL7_*ZNF2 and* P_GAL7_*ZNF2 pum1*∆ strains after transcriptional shutoff (YPD). The hybridization level of the ZNF2 was normalized to the rRNA levels to determine the change in transcript stability over time.

One reason that Pum1 de-repression alone may not be sufficient for filamentation is the absence of production of Znf2, the master regulator of hyphal morphogenesis. Overexpression of Znf2, a C_2_H_2_ Zinc finger transcription factor, can drive the yeast to hypha transition independent of environmental cues, thereby generating a homogeneous hyphal population^7^. Previous studies suggested that overexpression of *ZNF2* could not bypass the requirement of Pum1 for filamentation in *C. neoformans*^7^. To investigate this further, we generated a strain of *C. neoformans* that overexpresses *ZNF2* from the *GAL7* promoter (P_GAL7_*ZNF2*), resulting in glucose repressible and galactose inducible expression. As reported previously, overexpression of *ZNF2* in the wild type results in a uniformly filamentous population, whereas deletion of *PUM1* (P_GAL7_*ZNF2 pum1*∆) in the same strain abrogates filamentation (Fig. 4a). We then assessed the level of *ZNF2* overexpression in these two strains by northern blot in a time course of galactose induction. To our surprise, *ZNF2* induction was severely attenuated in the absence of *PUM1,* achieving less than 10% (9.33 ± 0.88%, n = 3) of the P_GAL7_*ZNF2* in the wild type background (Fig. 4b). Assessment of induction of the *GAL7* gene revealed equivalent induction between the two strains (Fig. 4b), ruling out an effect of Pum1 on the *GAL7* promoter.

Because the induction of *GAL7* was unaffected by *PUM1* deletion, we suspected that the effect of Pum1 on the *ZNF2* mRNA was post-transcriptional, and so we assessed the stability of *ZNF2* in *P*_*GAL7*_*ZNF2* versus its *PUM1* deletion strain *P*_*GAL7*_*ZNF2 pum1*∆. We grew the *P*_*GAL7*_*ZNF2* strain and its *PUM1* deletion strain *P*_*GAL7*_*ZNF2 pum1*∆ to mid-log in YP-Gal (*ZNF2* inducing conditions) and then chased for 30 minutes after shifting to YPD (*ZNF2* repressing conditions). We found the induced *ZNF2* mRNA to decay with a half-life of T_1/2_ >30 minutes (~40.7) in the wild type P_GAL7_*ZNF2* (Fig. 4c). However in the *PUM1* deletion strain, P_GAL7_*ZNF2 pum1*∆, *ZNF2* mRNA decayed around 50% within 7 minutes of the shift from YP-gal to YPD (Fig. 4c), suggesting that Pum1 stabilizes *ZNF2*. The stability plots exhibiting the same trend of *ZNF2* decay were generated when the hybridization signal of *ZNF2* was normalized to actin probed under same conditions, shifting from YP-gal to YPD (Supplementary Fig. S2). Although *ZNF2* has a P3E in its 5′ UTR, we believe that the stabilization effect of Pum1 on *ZNF2* mRNA is occurring indirectly as the 5′ UTR of *ZNF2* is not part of the *P*_*GAL7*_*ZNF2* construct.

### Alternative transcription start site utilization relieves *PUM1* auto-repression during filamentation

**Figure 5:**
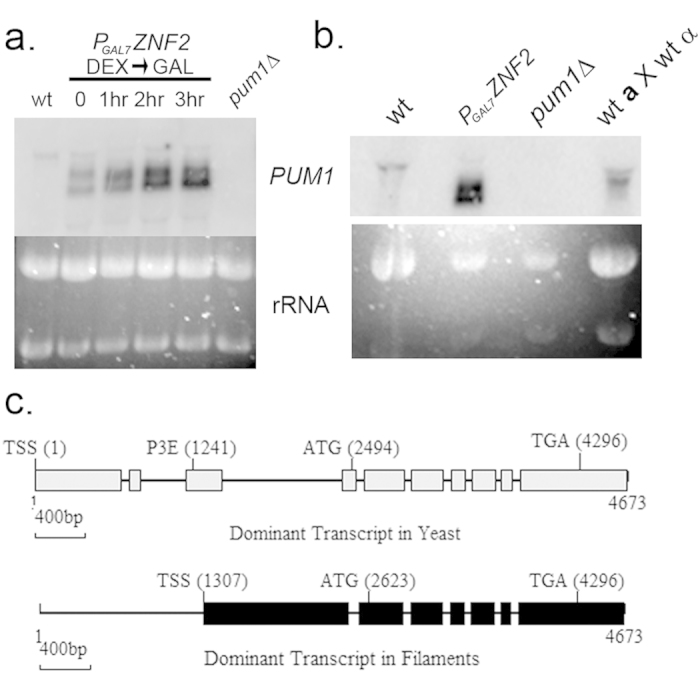
Relief of Pum1 translational auto-repression via an alternative transcription start site. (**a**) Northern analysis of *PUM1* expression in the P_GAL7_*ZNF2 s*train after induction. (**b**) Northern analysis of *PUM1* expression in wildtype (mid-log YPD), P_GAL7_*ZNF2* (3 hr induction), *pum1*∆ and wildtype mating RNA. (**c**) Schematics of the dominant transcripts in yeast and filaments identified by RACE and RT-PCR. Gray boxes represent the *PUM1* exonic sequences as annotated by the Broad Institute *Cryptococcus neoformans var. grubii* H99 Database. Black boxes represent exons of the alternative *PUM1* transcript predominant in the filaments. Lines represent intronic sequence. TSS stands for Transcription Start Site, P3E is Pum1/Puf3 binding element.

*PUM1* is a transcriptional target of Znf2^7^. To verify that in a P_GAL7_*ZNF2* strain, *PUM1* expression was induced, we analyzed *PUM1* transcript abundance in the P_GAL7_*ZNF2* background in a time course following a shift from dextrose to galactose. To our surprise, we observed the appearance of two smaller bands in the P_GAL7_*ZNF2* lanes in addition to the larger band seen in the wild type control that hybridized to a probe in the *PUM1* RNA binding domain coding sequence (Fig. 5a). To determine if these bands encoded alternative transcripts, we performed 5′ RACE using c-DNA from P_GAL7_*ZNF2* and two prominent bands were observed (SupplementaryFig. S3a). Sequence analyses of these bands revealed that the smaller amplicon began 60 bp downstream of the P3E. This suggests that Znf2 may be driving expression of *PUM1* from an alternative transcription start site. The Znf2-driven transcript does not include the P3E in its 5′ UTR, thus alleviating Pum1 auto-repression under conditions that require abundant Pum1 protein.

The identification of a shorter mRNA that begins downstream of the 5′ UTR P3E explains how under conditions of filamentation, Pum1 auto-repression is relieved. However, it does not account for the appearance of two shorter transcripts in the northern blot analysis. Immediately downstream of this alternative transcription start site, there is a 1 kb intron in the *PUM1* 5′ UTR. Because we estimated the size difference of the two smaller transcripts to be close to 1 kb, we asked if this 1 kb intron was retained in mRNA from filaments. By RT-PCR, we were able to detect the presence of this intron from cDNA under conditions during which the no-RT controls yielded no amplicon (Fig. S3b). To verify that this retained intron was amplified from cDNA, we utilized a forward primer within the 1 kb intron and a reverse primer downstream of the adjacent 50 bp intron. Sequencing of this amplicon revealed that both (1 kb and the adjacent 50 bp) the introns are retained (Fig. 5c). Indeed, analysis of the RNA seq reads used to annotate the genome of the wild type H99 strain does support retention of this 1 kb intron in a population of transcripts (http://www.broadinstitute.org)^13^. Additionally, a part of the ORF that lies between these two introns and encompasses the annotated start site is spliced suggesting that alternative splicing is taking place under filamentation conditions. This explains the appearance of two transcript species produced under conditions of Znf2 overexpression which differ in the retention or splicing of the mentioned introns. Also, the translation overview of the differentially spliced transcript suggests that the translation start site in the alternatively spliced transcript is 128 bp downstream of the annotated start site of *PUM1*, thereby generating a truncated version of Pum1 protein. Downstream of the alternative translation start site, the cDNA sequence from the filaments aligned perfectly with the annotated sequence suggesting that there are no other variant splicing events apart from what we have reported above.

Because P_GAL7_*ZNF2* is a synthetic construct being expressed in the wild type, we went on to determine if multiple *PUM1* transcripts were detectable in RNA isolated from fusant filaments obtained from mating of marked strains of wild type mating partners. Northern blot did reveal the presence of a smaller transcript under mating conditions that appears to align with the larger band of the doublet seen in *P*_*GAL7*_*ZNF2* (Fig. 5b). This would suggest that under mating conditions, the 1 kb 5′ UTR intron is retained in majority of *PUM1* transcripts, at least at the time point assayed.

**Figure 6:**
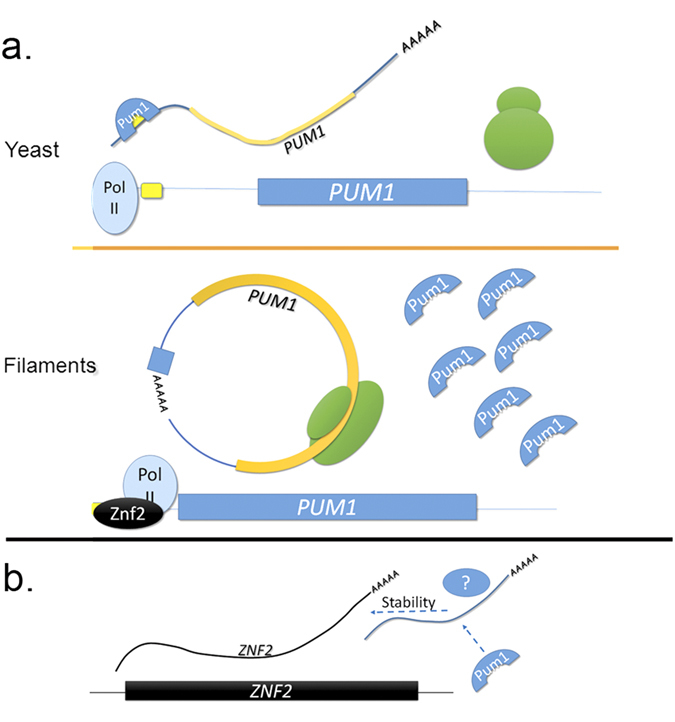
Model representing the morphotype specific auto regulation of Pum1. (**a**) In yeast cells, Pum1 binds to the P3E in its own 5′ UTR, repressing further translation of Pum1 protein (top). In filaments, *PUM1* transcription is driven by Znf2 producing an alternative mRNA that excludes the P3E, and is therefore not subject to Pum1 auto-repression (bottom). (**b**) Pum1 positively regulates *ZNF2* mRNA stability through an indirect mechanism, possibly by regulating an unknown factor that directly confers stability to *ZNF2*.

In this study, we demonstrate a novel role for a PUF family member in auto-regulation leading to morphotype-specific translational repression via an element located atypically in the 5′ UTR (Fig. 6a). This is the second report of a *C. neoformans* PUF protein interacting with a 5′ UTR element, as Puf4 interacts with the 5′ UTR of the ER stress transcription factor mRNA, *HXL1,* to regulate its splicing^9^. This may suggest that PUF protein regulation in *C. neoformans* will be fundamentally different than that of *S. cerevisiae,* in which most PUF elements are located in the 3′ UTR^8^. We also demonstrate that relief of *PUM1* auto-repression is insufficient to initiate the program of filamentous growth in the absence of the filamentation regulator Znf2. It is possible, however, that there are other developmentally-regulated Pum1 targets either repressed in yeast cells or regulated at the level of stability or localization in hyphae. Future studies will identify the morphotype-specific targets of Pum1. We expect to find among those targets factors that participate in the sexual development program, defining a *C. neoformans* filamentation RNA regulon, as the untranslated regions of functionally related transcripts often contain common *cis* elements^14^.

Under conditions of filamentation, Pum1 is required to support the stability of *ZNF2* mRNA. Because the expression construct used in this study lacks a P3E in its transcript, we hypothesize that Pum1 may be required to regulate the factor that directly influences *ZNF2* mRNA stability (Fig. 6b). Puf proteins can function as scaffolds or platforms for other RNA/protein binding partners which in turn regulate the fate of target transcripts^1^. It is possible that unknown Pum1 interacting proteins or mRNAs mediate *ZNF2* stabilization. Future studies will be aimed at the identification of other protein/RNA interacting partners of Pum1 that might be involved in Pum1-mediated post-transcriptional regulation.

At first glance, the production of *PUM1* transcript in yeast cells while simultaneously repressing its translation appears wasteful and inefficient. One of the proposed functions of filamentation in fungi is to allow the organism to escape a hostile environment both by foraging for nutrients and producing spores that can be dispersed in the environment. It is possible that the repression of Pum1 in yeast cells could allow for a rapid production of Pum1 protein in the event of a transient encounter with a mating partner in the environment, or under conditions where filamentation is optimal for nutrient foraging. This concept of readiness for filamentation is supported by studies of mating pheromone production in *C. neoformans*^15^. The mRNA encoding the mating pheromone is transcribed at extremely high levels in yeast cells, but that mRNA is immediately degraded^15^. The same mRNA then becomes stable under mating conditions. It is possible that other mechanisms, such as post-translational modification of Pum1, could elicit Pum1 de-repression even in the absence of Znf2. Continued investigation into mechanisms of Pum1 de-repression could reveal a role for environmental conditions priming yeast cells for the initiation of sexual development promoting escape, environmental dissemination and survival.

## Materials and Methods

### Generation of deletion and tagged strains

Parental strains used in this study are *C. neoformans* var. *grubii* strain H99, a non-passaged, fully virulent H99 gifted from Peter Williamson (UIC, NIAID) from an original stock derived from H99O gifted by John Perfect, Duke University. KN99**a** is a gift of Joe Heitman (Duke University)^13^. A complete list of strains used in this study is included in supplementary Table S1. *C. neoformans pum1*Δ **a** and α strains were constructed as described previously using NAT resistance cassette via homologous recombination^16^. The mutant strains were verified by northern and Southern blot. Complementation of *pum1*Δ **a** and α strains was performed by inserting *PUM1* gene with 1000 bp upstream and downstream cloned adjacent to the neomycin resistance cassette (Jennifer lodge, Washington University) in pJet-Neo to create pJetNEO-*PUM1*. The construct was transformed into *pum1*Δ **a** and α as described previously^17^. To generate myc tagged *PUM1*, the plasmid pJetNEO-*PUM1* was subjected to site-directed mutagenesis using the QuickChange XL site-directed mutagenesis kit (Agilent), using primers *PUM1*-myc F and *PUM1*-myc RC (Supplementary Table S2). The myc tag was inserted immediately upstream of the stop codon to create pJetNEO-myc*PUM1*. To generate mCherry tagged *PUM1,* PCR amplified *PUM1* (from genomic DNA of H99) and amplified mCherry (from pLk25, gift from Lukasz Kozubowski, Clemson University) were subjected to overlap extension PCR using primers as listed in Table 1 to generate *PUM1*mCherry fusion product. This fusion product was cloned in pJet-Neo to create pJetNEO-mCherry*PUM1. In vitro* site-directed mutagenesis was performed to mutate the Pum1 consensus element (P3E) in 5′ UTR of *PUM1* using the QuickChange XL site-directed mutagenesis kit (Agilent). To mutate TGTA of P3E at 547 position in pJetNEO-myc*PUM1*, primers EMSDM F and EMSDM RC as listed in Table S2 were used that generated pJetNEO-myc*PUM1EM* construct. To mutate the RNA binding domain (RBD) of *PUM1*, three residues in the seventh repeat of *PUM1* (required for binding); S351, N352 and E355 were mutated to alanine by site directed mutagenesis in pJetNEO-myc*PUM1* using primers RBDSDM F and RBDSDM RC as listed in Table S2. All the constructs generated were introduced into *pum1*Δ **a** and α using biolistic transformation^17^. To create Znf2 overexpressing strain, PCR amplified Znf2 was fused with Gal7 promoter and ligated into PBS-NAT vector. The resulting construct was then biolistically introduced in H99 thereby generating P_GAL7_*ZNF2.* Next, *PUM1* was knocked out from this overexpresser strain generating P_GAL7_*ZNF2 pum1*∆.

### RNA Isolation, Stability time course and Northern Blot Analyses

Mating spots from V8 agar plate were scraped off and subjected to mechanical disruption as described previously^18^. For transcript stability analyses, P_GAL7_*ZNF2* and P_GAL7_*ZNF2 pum1*∆cells were grown to mid-log in YP galactose (induction), washed and resuspended in 30 ml of YPD thereby repressing the GAL7 promoter. Five-milliliter aliquots were harvested every 15 min for 1 h and stored at −80 °C until RNA extraction. Northern blot was performed as described previously^18^. Hybridization signal was quantified using Quantity One software and normalized to the signal of the ribosomal bands. Graphpad prism software was used to fit the data to the least squares non-linear regression model. mRNA half-life was calculated from the slope of the regression line. Data that generated distinct regression lines with a *p* value less than 0.05 were deemed statistically significant.

### Fluorescence Microscopy

To detect localization of *PUM1*, filaments were scraped off from bilateral mating spots of mCherry complemented *pum1*Δ. The filaments along with some yeast cells were spotted on glass slides mounted with glycerol. Z-stack images were captured in RFP and TL-DIC channels using a Leica AM TIRF MC-fluorescence microscope. Image analysis and de-convolution was performed using LAS-AF software.

### Western Blot Analyses

H99, rpb4∆::RPB4myc, αpum1∆::*PUM1*myc and αpum1∆::*PUM1*P3EΔmyc were grown to mid-log in YPD at 30 °C. Cultures were harvested, washed with sterile deionized water, and whole cell lysates were obtained by mechanical disruption as described previously^18^. Western blot analysis was performed using primary mouse anti-myc antibody followed by secondary IR680-conjugated Donkey anti-mouse antibody as described previously^18^.

### Protein Expression and Purification

A truncated *PUM1* sequence containing the pumilio RNA binding domain was inserted into the pET14B vector and then transformed into *Escherichia coli* BL21(DE3)pLysS^9^. The recombinant protein product was purified using nickel agarose column affinity purification (Qiagen). Protein lysate was dialyzed and stored in 50 mM PO_4_, pH 8, 250 mM NaCl, and 5% glycerol at 4 °C^9^. Expression products were resolved on SDS PAGE followed by simple blue staining.

### Native RNA EMSA

The labeled RNA probe consisting of 22 bp of the *PUM1* 5′ UTR containing the cis element, labeled at the 5′ end with TYE-705 was obtained from IDT. Binding reactions were carried out in 20 μL volumes containing 10 μg of protein, 0.5 pmol of labeled RNA probe, competitor RNA (5x, 50x molar excess), and mutated competitor RNA in binding buffer^9^. Reactions were incubated for 25 minutes at room temperature. The 20 μL reactions were loaded onto 6% DNA retardation gel and run with 1x TBE running buffer at 100V. Probe mobility was detected on a LI-COR Odyssey Infrared Imaging System.

### Race and RT-PCR

5′ and 3′ *PUM1* RACE was performed from H99, and 5′ *PUM1* RACE was performed from P_GAL7_*ZNF2* using GeneRacer^®^ Kit with SuperScript^®^ III RT and TOPO TA Cloning^®^ Kit for Sequencing (ThermoFisher Scientific). RNA from midlog H99, P_GAL7_*ZNF2* induced for 3 hours in YP-Gal and mating culture from V8 plate was isolated and treated with DNase, and then cDNA was generated using an iScript cDNA synthesis kit^9^. Reaction mixtures lacking reverse transcriptase were included as a control for DNA contamination. The cDNA was used in a semi quantitative PCR. The two dominant bands obtained were further cloned in pJet1.2 blunt as per the manual (CloneJET PCR Cloning Kit) and subsequently sequenced using pJet1.2 forward and reverse primers.

## Additional Information

**How to cite this article**: Kaur, J. N. and Panepinto, J. C. Morphotype-specific effector functions of *Cryptococcus neoformans PUM1. Sci. Rep.* **6**, 23638; doi: 10.1038/srep23638 (2016).

## Supplementary Material

Supplementary Information
