# Supplementary material for: Morphotype-specific effector functions of Cryptococcus neoformans PUM1
Source: Sci Rep. 2016 Mar 24;6:23638. doi: 10.1038/srep23638 (PMC4806291; doi:10.1038/srep23638)
Supplement: Supplementary Information [file srep23638-s1.pdf]

Supplementary Information

Morphotype-specific effector functions of *Cryptococcus neoformans PUM1*

Jan Naseer Kaur and John C. Panepinto*

Department of Microbiology and Immunology, Witebsky Center for Microbial Pathogenesis and Immunology, Jacobs School of Medicine and Biomedical Sciences, University at Buffalo, the State University of New York

Supplementary Material:

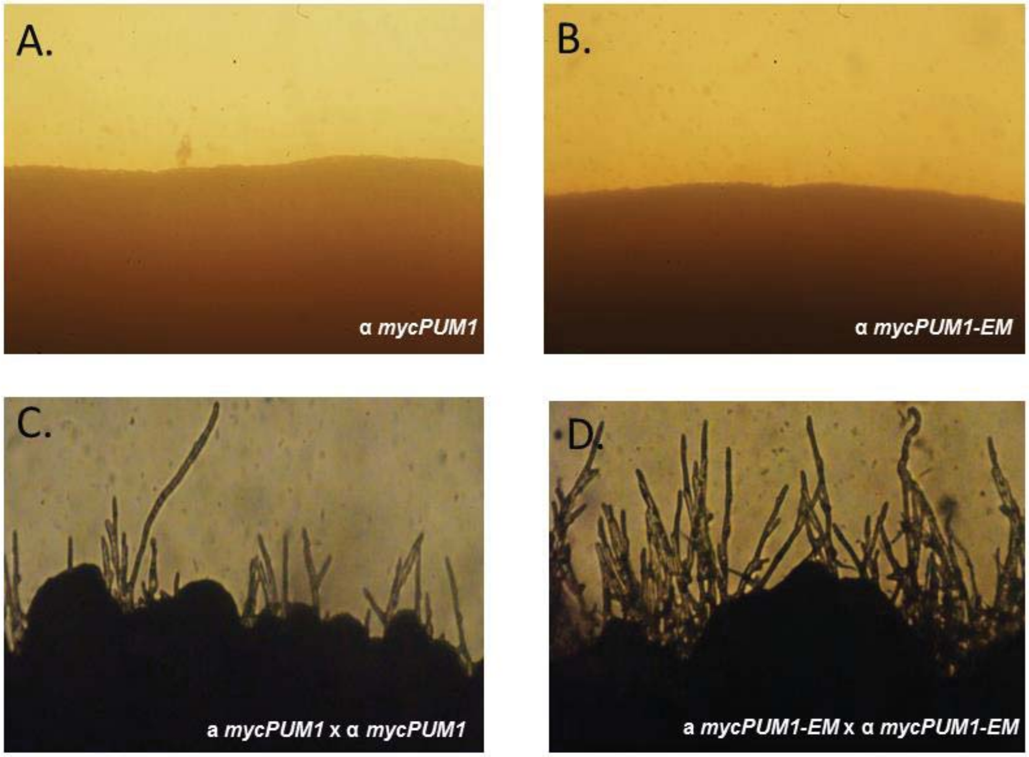

Fig. S1: De-repression of *PUM1* is not sufficient to induce filamentation in the absence of opposite mating partner.

Equal number of α mating type alone (A and B) and co-culture of α and **a** mating partners (C and D) of *mycPUM1* (A and C) *and mycPUM1-EM* (B and D) were spotted on to the V8 mating media for 72 hours in dark at room temperature. Filamentation was assessed by light microscopy at 100X magnification.

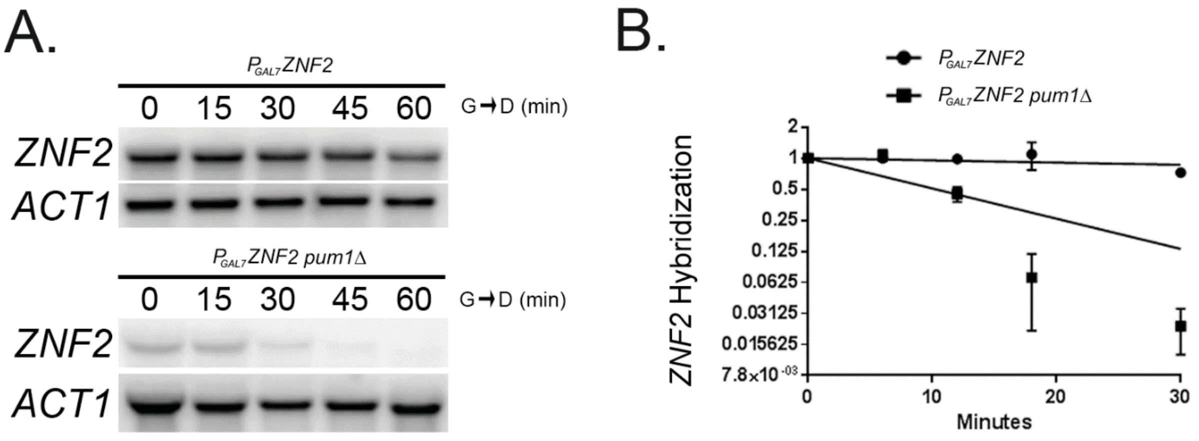

Fig. *S2*: *PUM1* positively regulates the stability of *ZNF2*

(**a**) Northern analysis of *ZNF2* stability in the P_{GAL7}*ZNF2 and* P_{GAL7}*ZNF2 pum1Δ* strains after transcriptional shutoff (YPD). The hybridization level of the ZNF2 was normalized to the actin levels to determine the change in transcript stability over time. (**b**) Log 2 plot of relative expression of *ZNF2* transcript normalized to the actin hybridization was used to generate the decay curve. The graph shown is the representative of two biological replicates.

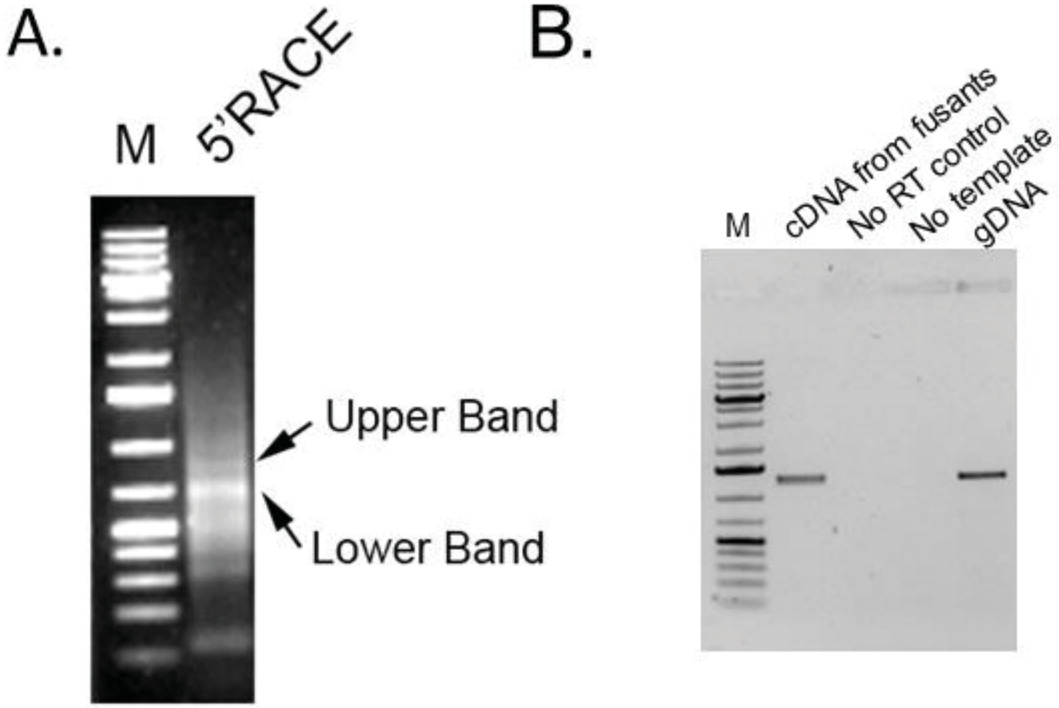

Fig. S3: Analysis of alternative *PUM1* mRNA

(**a**) 5' *PUM1* RACE was performed from midlog culture of P_{GAL7}*ZNF2* induced for 3 hours in YP-Gal using GeneRacer® Kit. The RACE-PCR product was subjected to agarose gel electrophoresis. The two dominant bands obtained, marked with the arrows, were excised from the gel, purified and cloned into pJET for conventional sequencing. (**b**) RT-PCR from the cDNA transcribed from wild type mating RNA was performed using a forward primer within the 1 kb intron and a reverse primer downstream of the adjacent 50 bp intron. The size of the amplicon obtained from the mating cDNA (lane1) was comparable to that of the gDNA (lane4) suggesting that the 1kb intron is retained. No amplification was detected in the no-RT (lane2) and no-template (lane3) controls.

TABLE S1: STRAINS

H99	Peter Williamson, University of Illinois at Chicago/NIAID
KN99	Joe Heitman, Duke University
$H99^{NAT}$	Joe Heitman, Duke University
$KN99^{NEO}$	Joe Heitman, Duke University
α *pum1Δ*	This study
**a** *pum1Δ*	This study
α pum1Δ Nat	This study
**a** *pum1Δ Neo*	This study
α pum1Δ::mChPUM1	This study
**a** *pum1Δ::mChPUM1*	This study
α pum1Δ::mycPUM1	This study
**a** *pum1Δ::mycPUM1*	This study
α pum1Δ::RBDΔmycPUM1	This study
**a** *pum1Δ::RBDΔmycPUM1*	This study
α pum1Δ::EM mycPUM1	This study
**a** *pum1Δ::EMmycPUM1*	This study
P_{GAL7}*ZNF2*	This study
P_{GAL7}*ZNF2 pum1Δ*	This study

TABLE S2: PRIMERS

NAME	SEQUENCE
PUM1 KO 5' Arm F	TAATAATCTAGACCAGCTTATCACCGAATTGC
PUM1 KO 5' Arm RC	TAATAAAGATCTGCAATAGCCATCTCCTGC
PUM1 KO 3' Arm F	TAATAAGGTACCCAAAGCTGCATCCCTGGG
PUM1 KO 3' Arm RC	TAATAACTCGAGCCTCCTCGTCGAACTCACC
PUM1 Myc F	CATTGAAGGGTGGGAGCAGAAGCTTATCTCTGAGGAGGACCTTTAGTGATTAATCTCTCG
PUM1 Myc RC	CGAGAGATTAATCACTAAAGGTCCTCCTCAGAGATAAGCTTCTGCTCCCACCCTTCAATG
PUM1 mCherry F	AGGACGACATTGAAGGGTGGATGGTGAGCAAGGGCGAGG
PUM1 mCherry RC	TAATAATCTAGACCCTCTTCACGTGGACGC
EM SDM F	GAAGGGATTGGGCAAACGACAAAACATACGCATTCGGACTCTC
EM SDM RC	GAGAGTCCGAATGCGTATGTTTTGTCGTTTGCCCAATCCCTTC
PUM1 RBD SDM F	CGACACAAATTCGCCGCCGCCGTCGTTGCCAAGGCCCTCATTCACG
PUM1 RBD SDM RC	CGTGAATGAGGGCCTTGGCAACGACGGCGGCGGCGAATTTGTGTCG
PUM1 Rec. RBDp F	TAATAAGGATCCTATGTCTCAATATCCGGGTAGGC
PUM1 Rec. RBDp RC	TAATAAGGATCCTTACTCGTTCTCCATCTGGGC
PUM1 F	CTGGTGTTGGTCGGGAGC
PUM1 RC	CCGTATACCACACTGCAGCC
PUM1 RACE 5' end	CGAGCTTCTGCTGGATAAATCG
PUM1 RACE 3' end	GAAGGACGACATTGAAGGG
ZNF2 Not1 F	TAATAAGCGGCCGCGCCAGCTTTAGCTCAAAATCG
ZNF2 Sal1 RC	TAATAAGTCGACGGAAGTCTTATTATGGGCGAGG
RT PCR F	GGGGTGTGGTGGGTTCG

RT PCR RC	CGACGAAATGAGGGGCAGG
